# Attitudes towards Futile Treatments, Moral Distress and Intention to Leave Job in Nurses

**DOI:** 10.1192/j.eurpsy.2023.399

**Published:** 2023-07-19

**Authors:** D. S. Kasım, F. Oflaz

**Affiliations:** ^1^Nursing, Acibadem University; ^2^Nursing, Koç University, Istanbul, Türkiye

## Abstract

**Introduction:**

The fact that nurses do not have a voice in the treatment decision of patients and that there is no framework about futile treatments can cause some psychological problems such as depression, burnout and moral distress (Yildirim et al., 2018). If not managed properly, moral distress leads to decreased job satisfaction, increased nurse turnover rates and intent to change the working area or leave the profession (Vieira & Doedato & Mendes, 2021).

**Objectives:**

This study aimed to explore the nurses’ attitudes towards futile treatments and its relationship with the moral distress, intention to leave the job and the other personal factors.

**Methods:**

This study has a descriptive and correlational design, carried out with 425 nurses, between April-May 2021 in Istanbul. The data were collected using a Personal Information Form, The Nurses’ Attitudes Towards Futile Treatment Scale(NATFTS), Moral Distress Scale(MDS) and Intention to Leave Scale (ILS). Personal Information Form: The form consists of 22 questions including the socio-demographics and professional characteristics. The Nurses’ Attitudes Towards Futile Treatment Scale (NATFTS): The scale was developed by Yildirim et al. in 2019, consisting of 18 items and uses a 5-point likert type scale.Moral Distress Scale(MDS):The scale, developed by Hamric (2012), adapted to Turkish by Karagözoğlu et al. (2017), consisting of 21 expressions, was designed to measure the level of moral distress in nurses. Intention to Leave Scale (ILS):The scale was developed by Wayne et al. (1997), and adapted to Turkish by Avcı ve Küçükusta(2008) in the form of 5 items. The data were analyzed by using descriptives, Kruskal Wallis test, Independent Samples t- test and ANOVA, Pearson Correlation analysis on SPSS 25.0 for Windows. For significance, p < .05 and 95% CI were assumed in the data analysis.

**Results:**

A moderate negative correlation was found between NATFTS score and the ILS score (r=-0.356, p<0.001). Nurses who think that futile treatments should be applied under the strict rules have less tendency to leave their jobs. On the other hand, there was a lower positive level of correlation between NATFTS score and the MDS score (r=0.295, p<0.001). That is, nurses who think that futile treatments should be applied under the strict rules have a higher level of moral distress. A low-level negative of relationship was found between the MDS score and the ILS score (r=-0,260, p<0,001). As the level of moral distress increases, the tendency to leave work decreases.

**Image 2:**

**Figure:**
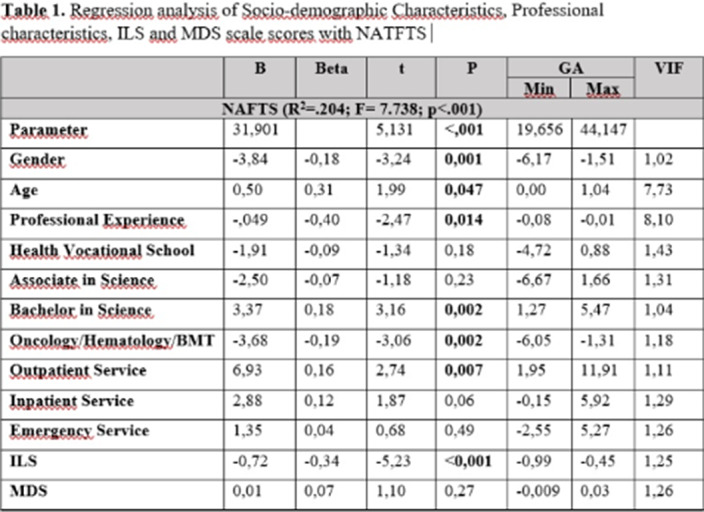

**Conclusions:**

It is believed that educating nurses and strengthening them psychologically will prevent them from experiencing moral distress, increase job satisfaction and reduce intention to leave (Cicolini et al., 2014). Considering the negative effects of ethical dilemmas about futile treatments on health professionals, it is recommended that legal regulations be made on the subject and institutions determine their own protocol.

**Disclosure of Interest:**

None Declared

